# Beyond Radical Emitters: Hot‐Exciton Electroluminescence From Singlet Diradicaloids

**DOI:** 10.1002/anie.6379738

**Published:** 2026-09-22

**Authors:** Mario Prosa, Eugenio Lunedei, Michele Orza, Davide Mesto, Roberto Cantoni, Francesco Reginato, Angela Punzi, Arthur R. J. Barreto, Yasi Dai, Benedetta Carlotti, Fabrizia Negri, Stefano Toffanin, Davide Blasi

**Affiliations:** ^1^ Istituto per lo Studio dei Materiali Nanostrutturati Consiglio Nazionale delle Ricerche Bologna Italy; ^2^ Dipartimento di Chimica “Giacomo Ciamcian” Università di Bologna Bologna Italy; ^3^ Dipartimenti di Chimica Università degli Studi di Bari Aldo Moro Bari Italy; ^4^ Department of Chemistry Biology and Biotechnology University of Perugia Perugia Italy; ^5^ Center for Chemical Catalysis—C3 and INSTM UdR Bologna Alma Mater Studiorum—Università di Bologna Bologna Italy

**Keywords:** electroluminescence, hot exciton, singlet diradicaloids, thiele hydrocarbon, transistors, trityl radicals

## Abstract

Overcoming the 25% spin‐statistical limit of fluorescent emitters remains a central challenge in organic electroluminescence. Although radical emitters efficiently harvest doublet excitons in organic light‐emitting diodes, their optoelectronic performance can be affected by limited charge‐carrier mobility and efficiency roll‐off. Here we introduce trityl‐based singlet diradicaloids with small diradical character as a new class of open‐shell emitters and demonstrate their operation in organic light‐emitting transistors (OLETs) using the polychlorinated Thiele hydrocarbon (**TTH**) as emitter. Comparison with previously reported radical‐based devices reveals spin utilization around or exceeding 0.38 in **TTH**‐based OLETs. Time‐resolved electroluminescence, fs and ns transient absorption measurements, and quantum‐chemical calculations demonstrate that this enhancement does not originate from triplet–triplet annihilation but from hot‐exciton mechanism enabled by the diradical electronic structure, where the T_2_ state efficiently repopulates the emissive singlet via reverse intersystem crossing (RISC). Unlike conventional hot‐exciton emitters based on donor–acceptor architectures, this system demonstrates hot‐exciton behavior in a singlet diradicaloid framework. Reduced charge trapping further highlights singlet diradicaloids with small diradical character as promising platforms for efficient and stable electroluminescent devices.

## Graphical abstract

The first hot‐exciton mechanism in a singlet diradicaloid is demonstrated in a polychlorinated Thiele hydrocarbon‐based organic light‐emitting transistor. Reverse intersystem crossing involving the T_2_ and bright singly excited states enables electroluminescence beyond the 25% spin‐statistical limit, leading to remarkable external quantum efficiency.

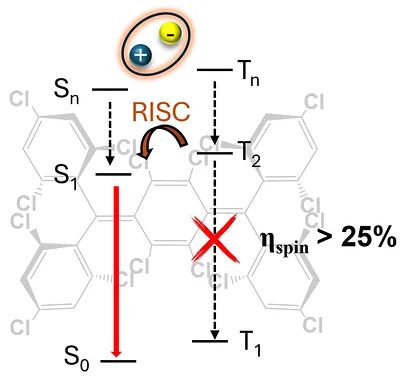

## 1 Introduction

Among the strategies to overcome the spin‐statistical limit in electroluminescent devices, luminescent free radicals are particularly attractive because their doublet spin multiplicity allows harvesting all electrically generated excitons, allowing 100% internal quantum efficiency [1, 2, 3]. Accordingly, highly fluorescent polyhalogenated trityl radicals have emerged over the past decade as efficient organic light‐emitting diode (OLED) emitters with excellent chemical robustness, enabling remarkable external quantum efficiencies (EQEs) in the far‐red to near‐infrared region [4, 5, 6].

Beyond conventional OLEDs, efficient emitters are also highly desirable for emerging electroluminescent devices such as organic light‐emitting transistors (OLETs), which combine charge transport, switching, and light emission within a single planar architecture [7]. The possibility of spatially controlling the micrometer‐sized emission zone along the channel enables compact device layouts, making OLET promising for integrated sensing and simplified display circuitry [8, 9, 10, 11, 12]. However, the scarcity of materials that simultaneously provide high charge mobility and strong electroluminescence remains a major bottleneck, limiting OLET performances compared with established OLED technologies [13, 14, 15]. In this context, luminescent radicals offer a promising solution. Recently, some of us demonstrated the first OLETs employing radical emitters, exploiting doublet excitons to enhance internal quantum efficiency [16]. Bilayer devices incorporating the 2,6‐dichloro‐4‐(2,6‐dimethoxyphenyl)phenyl (2,4,6‐trichlorophenyl)methyl radical (**PyBTM‐(Me_2_Res)** in Figure 1a) achieved EQEs up to 0.20% in the far‐red region. Yet radical‐based devices can suffer from EQE roll‐off, likely caused by exciton–charge annihilation induced by limited charge‐carrier mobility [1, 16, 17]. Specifically, charge trapping simultaneously governs radiative recombination and non‐radiative quenching in these systems [12, 18], thus new strategies for exciton formation and management are required. Although “hyperfluorescence” concepts combining TADF donors with radical acceptors can mitigate turn‐on voltage and efficiency roll‐off in OLEDs [19, 20], identifying intrinsically stable fluorescent materials that exceed the 25% spin‐statistical limit without complex device architectures remains a central challenge [21, 22].

**FIGURE 1:**
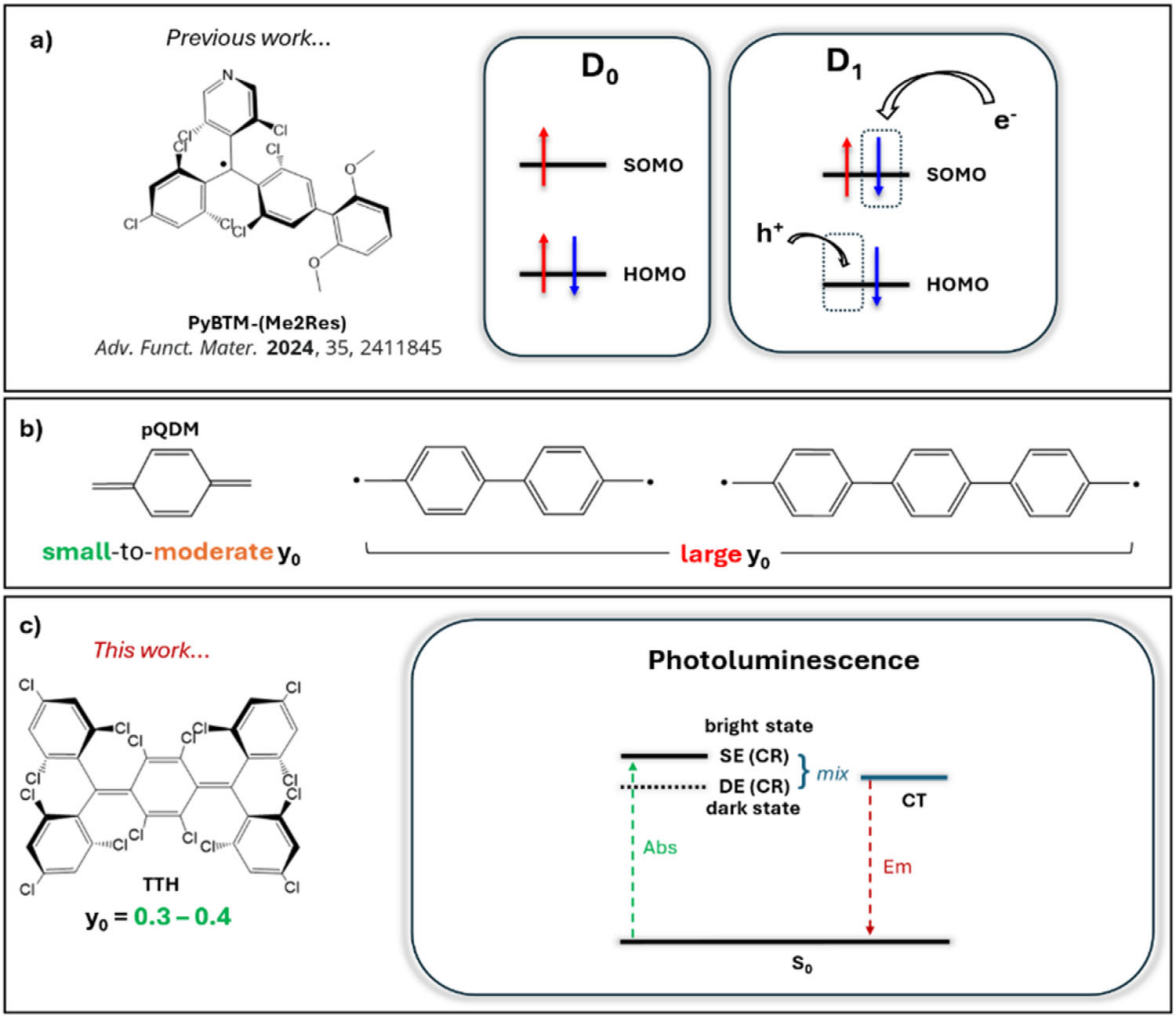
(a) Molecular structure of **PyBTM‐(Me_2_Res)**, previously adopted as an emitter in an OLET device. Thanks to its unpair electron in SOMO level, the molecule enhances charge trapping in electroluminescence formation. (b) Molecular structure of the para‐quinodimethane (pQDM) unit, typically used for the design of singlet diradicaloids. The diradical character can be tuned through the extension of the pQDM system. (c) Molecular structure of the **TTH** and description of its energetics, where the photoexcited SE state, and the nearly degenerate doubly excited (DE) state (both charge resonance) mix, leading to the formation of a highly emissive CT (zwitterionic) state.

In this context, singlet diradicaloids have attracted increasing attention. Trityl‐based para‐quinodimethane (pQDM) derivatives display tunable diradical character (*y*_0_), that grows with bridge length (*y*_0_ = 0 for a closed‐shell quinoidal species and *y*_0_ = 1 for a pure diradical) [23], enabling open‐shell electronic structures while retaining high stability [24, 25, 26, 27, 28, 29, 30, 31]. Hosting two unpaired electrons, diradicaloids exhibit richer photophysics than monoradicals; especially, derivatives with quasi‐degenerate singlet–triplet ground states and delocalized spin density, that is, systems with an extended pQDM bridge where *y*_0_ approaches the unitary value (Figure 1b), can show spin‐allowed transitions from both singlet and triplet excited states [32], although increasing diradical character in this class of pQDM‐based singlet ground state diradicaloids can be associated with reduced oscillator strength and diminished photoluminescence efficiency [28]. Consequently, a moderate diradical character offers an attractive compromise, preserving the open‐shell excited‐state manifold while maintaining efficient radiative decay and excellent chemical stability. In this context, shorter pQDM derivatives such as the polyhalogenated Thiele hydrocarbon (**TTH** in Figure 1c) combines moderate diradical character (*y*_0_ ≈ 0.32 – 0.44 [24]) with exceptional stability and high photoluminescence quantum yield (PLQY) even in the solid state, making it an attractive candidate for optoelectronics [33].

Its emissive state arises from mixing between a bright singly excited (SE) state and a dark low‐lying doubly excited (DE) state, both characterized by a charge‐resonance (CR) character, yielding a symmetry‐broken charge‐transfer (zwitterionic) state that couples a large transition dipole moment with pronounced charge‐transfer (CT) character (Figure 1c) [30, 34, 35]. The moderate diradical character of **TTH** is crucial because it introduces partial open‐shell character that strongly influence the excited‐state energy landscape. Unlike conventional closed‐shell chromophores, the richer manifold of diradical excited states can give rise to unconventional spin‐state ordering, including cases where higher‐lying triplet states are energetically close to or above emissive singlet states, that can participate in spin‐mediated excited‐state conversion [36, 37, 38]. Hence, the energetic ordering of the excited states in singlet diradicaloids, with T_1_ states lying closer to the ground state than to the lowest excited singlet (Δ*E*_T1‐S0_< Δ*E*_S1‐T1_) [37, 39], supports hot‐exciton pathways analogous to hybrid local/charge‐transfer (HLCT) systems, where higher‐lying triplets efficiently repopulate emissive singlets via reverse intersystem crossing (RISC) [40, 41]. Therefore, in **TTH**, the moderate diradical character does not merely determine the emissive mechanism itself, but rather creates the electronic conditions required for the formation of suitable excited‐state configurations and efficient spin conversion pathways. Together, the efficient emission and the unique excited‐state configuration, provide a clear rationale for singlet formation probabilities exceeding 25% while avoiding limited charge carrier mobility as expected when singly occupied molecular orbitals (SOMOs) in persistent radical enhance charge trapping. Here we validate this concept by employing **TTH** as the emitter in an n‐type OLET. Compared with **PyBTM‐(Me_2_Res)**, **TTH**‐based devices exhibit spin statistics around or exceeding 0.38 and relevant emitted optical power. Time‐resolved electroluminescence and ultrafast transient absorption measurements, together with quantum‐chemical calculations support a hot‐exciton mechanism involving the T_2_ and bright SE states, showing that trityl‐based pQDM singlet diradicaloids are less affected by charge trapping and surpass the conventional 25% singlet formation limit.

## 2 Result and Discussion

In Figure 2a we report the architecture of the unipolar bilayer OLET designed to host the diradicaloid **TTH** molecule as light‐emitting material. The emissive layer (EML) comprised 2,2′,2′′‐(1,3,5‐benzinetriyl)‐tris(1‐phenyl‐1‐H‐benzimidazole) (TPBi) as the matrix in which **TTH** is dispersed [42]. As electron‐transporting organic semiconductor (OSC), namely N,N’‐ditridecylperylene‐3,4,9,10‐tetracarboxylic diimide (P13) was selected for its excellent electron mobility of 0.25 cm^2^V^−1^s^−1^ [16, 43, 44], while polymethyl methacrylate (PMMA) was used as dielectric material due to the high‐quality of the P13/PMMA interface which ensures proper charge transport [45]. To favor the exciton formation process in the EML, the device architecture comprised also a hole injection layer (HIL), namely 4,4′‐bis(N‐carbazolyl)‐1,1′‐biphenyl (CBP). In Figure 2b, a favorable energy alignment is observed between the lowest unoccupied molecular orbitals (LUMOs) of P13 (–3.89 eV) and **TTH** (–4.05 eV), which facilitates direct electron injection into the **TTH** sites during OLET operation. The introduction of CBP as a HIL is expected to promote efficient hole injection from the gold electrode into the emissive layer (EML), while simultaneously blocking electrons from reaching the drain electrode, owing to its high‐lying LUMO level (–1.87 eV). We have previously demonstrated the effective operation of this device architecture using the open‐shell **PyBTM‐(Me_2_Res)** molecule, whose energy levels are comparable to those of **TTH** [16, 24]. Therefore, replacing **PyBTM‐(Me_2_Res)** with **TTH** as the emissive compound is expected to yield similarly efficient OLET performance, thus the same device structure was retained for the present study. Complete OLET devices were fabricated by using two different doping concentrations of **TTH** within the EML, that is, 5% and 15%. The figures of merits of the resulting **TTH**‐OLETs are summarized in Table 1 while the electrical characteristics are reported in Figure S1.

**FIGURE 2:**
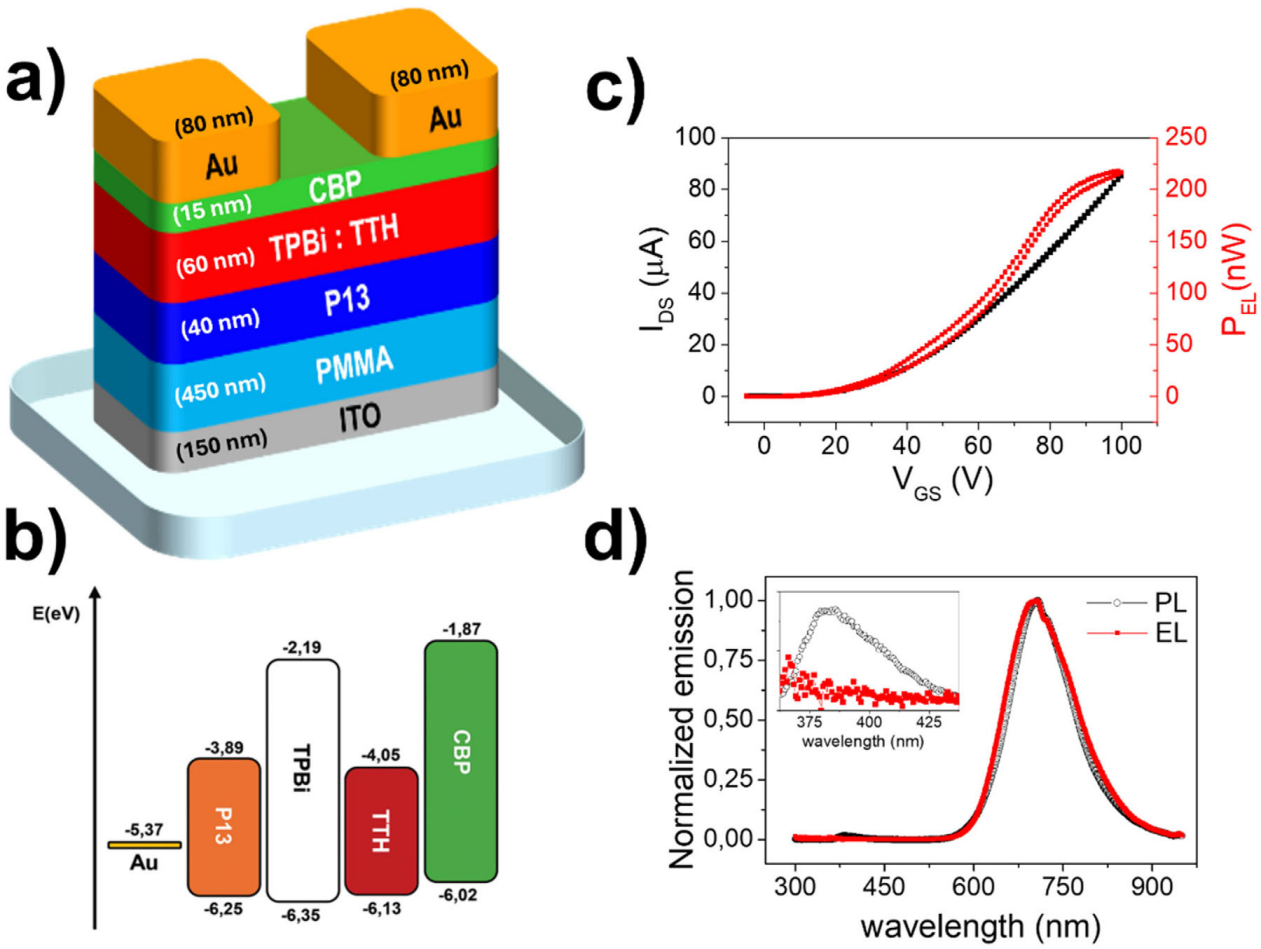
**TTH**‐OLETs: (a) schematic representation of the device architectures; (b) energy level diagram of the used materials; (c) (black) n‐type saturation transfer curve for a **TTH**‐OLET doped at 15%, *V*_DS_ = 100 V; (red) corresponding electroluminescence signal; (d) comparison between PL and EL spectra; d. inset) zoom of (d) between 375 and 425 nm.

**TABLE 1:** Summary of the figures of merits of **TTH**‐OLETs.

TTH conc. [%]	EML PLQY	*μ* ^a^ (cm^2^ V^−1^ s^−1^)	*V*_th_ ^a^ (V)	*I*_DS_ ^b^ (µA)	EL^b^ (nW)	EQE^b^ (%)
5%	0.43	2.3 ∙ 10^−2^	8.9	87	8	0.005
15%	0.35	1.7 ∙ 10^−2^	6.4	85	217	0.15

^a^ Calculated from saturation transfer curves in the saturation regime.

^b^ at *V*_GS_ = *V*_DS_ = 100 V.

Good electron‐transport behavior was obtained for both devices, with an electron mobility around 2 ∙ 10^−2^ cm^2^ V^−1^ s^−1^, a threshold voltage (*V*_th_) < 10 V, and a maximum drain current (*I*_DS_) close to 90 µA. Although both devices showed similar electrical behavior (Table 1), significant differences were observed in terms of light‐emitting characteristics. The optical emission power (*P*_EL_) is 217 nW for 15% of **TTH** doping of the EML, whereas a lower value (8 nW) was measured for OLETs based on 5% of **TTH** content. The increase in **TTH** concentration apparently boosts the optical figures of merits of the device, leading to a 30‐fold enhancement of *P*_EL_. The 15% doped **TTH**‐OLETs showed a remarkable EQE of 0.15% at *V*_GS_ = *V*_DS_ = 100 V (Figure S2 and Table 1). The content of **TTH** in EML was further increased to 22.5%, but the EQE of the corresponding OLET dropped to 0.05% (Table S1). We also evaluated various device structures by making slight modifications to the original design (Figure S3 and Table S2). While the decrease of the EML thickness affects the optical power of the device, the use of asymmetric layout of top electrodes allows the device to achieve higher source‐current and consequently maximize the brightness reaching a value as high as 7.63 cd m^−2^, thus a factor 10 higher with respect to the previous device layout.

The emissive behavior of **TTH**‐OLETs as a function of **TTH** concentration is in contrast with the observed PLQY of the corresponding solid‐state TPBi:**TTH** blends. 5% and 15% doped films exhibited PLQY values of 0.43 and 0.35, respectively. This hints that the host–guest (TPBi‐**TTH**) energy transfer process is more efficient at the lower **TTH** concentration. On the contrary, in the devices, where exciton formation is driven by charge injection, the device EQE was maximized by using 15% of **TTH**. This discrepancy suggests that, in 15% **TTH**‐OLETs, direct exciton formation on **TTH** sites via charge injection and transport on the TPBi matrix is the relevant process for electroluminescence. Indeed, on one hand, both photoluminescence and electroluminescence spectra exhibit a similar broad feature centered at *λ* ≈ 704 nm, associated to radiative deactivation of excited diradicaloid molecules, as typically observed in **TTH** solutions [24]. On the other hand, as shown in Figure 2d (inset), whereas a residual TPBi emission is detectable in the 375–425 nm range of PL spectrum, it is not present in the EL one. Given that energy transfer is the only possible mechanism in PL, the residual host emission arises from a suboptimal host–guest transfer rate. The absence of such high energy steady‐state feature in device under biasing highlights that energy transfer is not the dominant mechanism governing exciton generation in 15% doped **TTH**‐OLETs. To support this assumption, we collected transient EL from 5% and 15% doped **TTH**‐OLETs to get a more in‐depth knowledge of the photophysical processes at the basis of the light‐emission in devices.

Transient EL measurements (EL(t)) were carried out by biasing the device via a periodic rectangular *V*_DS_ pulse (100 V), while keeping *V*_GS_ fixed at 100 V and collecting the device emitted light at 700 nm (maximum of EL spectrum), implementing the same experimental methodology reported elsewhere [16, 44]. Figure 3a shows the EL(t) signal of a 5 % doped **TTH**‐OLETs (*V*_DS_ pulse width  =  1µs). Within a single *V*_DS_ pulse, EL(t) intensity was observed to have a fast onset followed by a gradual increase without any remarkable change in spectral profiles (Figure 3a inset). The signal collected at the end of *V*_DS_ pulse can be fitted by tri‐exponential EL(t) having an intensity‐averaged decay time of 0.7 ± 0.1 µs, that is dominated (weight 56 %) by a *τ*_1_ ∼ 1.1 µs (Table S3). Time‐resolved photoluminescence measurements were performed on both isolated **TTH** in solution, **TTH** in PMMA matrix and **TBPi**:**TTH** thin films (Figures S11–S15). **TTH** dispersed in PMMA exhibits a photoluminescence lifetime of approximately 10 ns, independent of the excitation wavelength and similar to the solution [24], indicating that direct excitation of **TTH** always relaxes through the same emissive pathway without the involvement of long‐lived excited states. In contrast, when **TTH** is dispersed in the **TBPi** host and only when the excitation selectively addresses **TBPi** in the UV, the **TTH** emission displays a much longer lifetime (hundreds of nanoseconds), demonstrating that a different excited‐state population pathway is operative under conditions relevant to device operation. The much longer EL decay time that can be assigned to **TTH** optical emission (*vide infra*) suggests that host‐to‐diradicaloid energy transfer, is not occurring in **TTH**‐OLETs or is overshadowed by other slower and predominant phenomena. As a result, any possible energy‐transfer pathways are effectively undetected in devices. It can be ruled out that the EL(t) dynamic during the *V*_DS _pulse is mastered by high value of device RC constant, as corroborated by the simultaneous fast evolution of the transient source‐drain current (Figure 3a, red trace). In order to identify possible emissive species, in operando time‐integrated EL(*λ*) spectra were collected within various time intervals during application of *V*_DS_ pulse (Figure 3a, inset).

**FIGURE 3:**
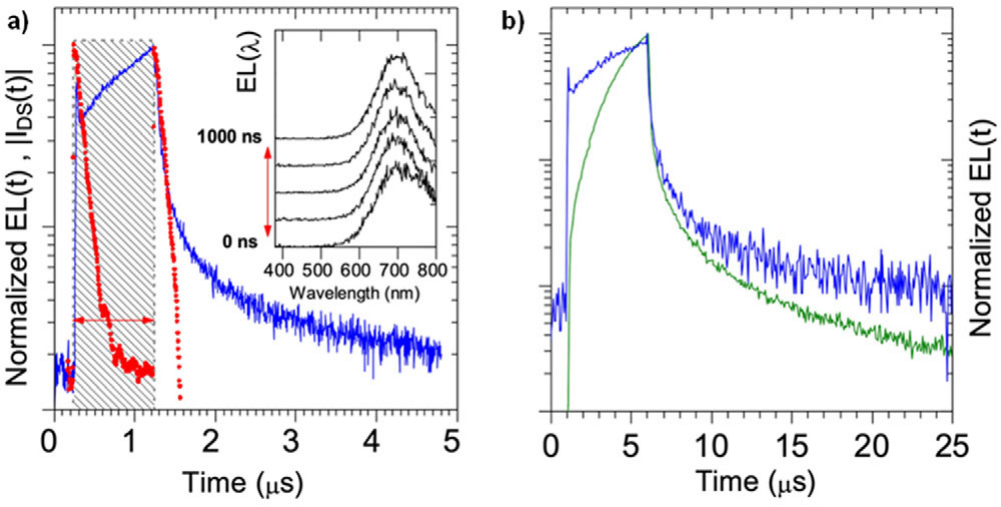
Transient electroluminescence EL(t) of **TTH**‐OLETs driven by constant *V*_GS _ =  100 V and by pulsed *V*_DS _ =  100 V. Panel (a) blue trace: normalized semi‐log plot of EL(t) as detected at *λ*  =  700 nm from a 5% doped **TTH**‐OLETs (1 µs‐long pulse and 5 µs‐wide time range), biasing the device by rectangular *V*_DS_ pulses (gray shaded area, width  =  1000 ns, 108 kHz rep. rate); red trace: normalized modulus of transient *I*_DS_. Panel a. inset: time evolution of EL spectrum, during the application of the voltage pulse, as integrated over 200 ns time intervals (curves are vertically shifted for sake of clarity). Panel (b) comparison between 5% (blue line) and 15% (green line) doped **TTH**‐OLETs: normalized semi‐log plot of EL(t) in a 25 µs‐wide time range.

EL(*λ*, Δ*t*) exhibited identical features, in every observed time intervals, which are similar to those of steady‐state PL spectrum of **TTH** only in the 600–800 nm emission region. This confirms that emissive excitons are formed on single diradicaloid sites. Transient EL(*t*) measurements were also carried out in longer time ranges, by increasing the *V*_DS_ pulse width from 1 µs to 5 µs. The observed time range was extended up to 100 µs (Figure S4a). Regardless of the increased pulse width, similar electroluminescence features were recorded apart from a slower time decay value (intensity‐averaged decay time of 4.7 ± 0.1 µs in Table S3). We can safely discharge the possibility of time‐delayed fluorescence generation at longer time due to triplet–triplet annihilation (TTA) as the monotonic decrease of the collected EL decays collected at a very long time‐range demonstrates (Figure S4a). In order to experimentally assess the triplet excited state energy of **TTH**, we performed sensitization experiments via nanosecond laser flash photolysis (Figures S16–S20 and Table S6) by employing triplet energy donors characterized by different triplet energies (*E*_T_) [46, 47]. A very efficient quenching of the sensitizer triplet lifetime in the presence of **TTH** was observed in the case of phenanthrene (*E*_T_ = 2.92 eV), anthracene (*E*_T_ = 1.85 eV), and tetracene (*E*_T_ = 1.27 eV) for which a quenching rate constant consistent with the diffusional rate constant was found. A less efficient quenching was obtained when using rubrene (*E*_T_ = 1.14 eV) and pentacene (*E*_T_ = 0.77 eV), indicating that in these cases the sensitizer triplet may be energetically closer to the T_1_ state of **TTH**. The evidence that quenching occurs with a diffusional rate constant also when an energy donor characterized by a high triplet energy (e.g., phenanthrene) is used, suggests the presence of an upper T_n_ excited state of **TTH**, which may correlate effectively with the sensitizer triplet. These evidences are in agreement with quantum‐chemical calculations that predict an adiabatic S_0_‐T_1_ energy difference, for **TTH,** of the order of 0.2 eV, and ΔE_S1‐T2_ = −0.24 eV (Figure S7 and Table S4, *vide infra*). Transient EL(*t*) measurements were carried out also on 15% doped **TTH**‐OLETs in the long time‐range (Figure S4b). With respect to 5% doped **TTH**‐OLETs, the presence of a higher amount of **TTH** in the EML did not induce any modification of the EL decay time of the devices (Table S3). A relatively slow EL(t) decay was also observed in OLETs implementing **PyBTM‐(Me_2_Res)** radical as emissive species dispersed in the same host matrix [16]. In that case, the kinetics of exciton formation and radiative deactivation was driven by dynamics of charge‐trapping and ‐release mechanisms at the radical emissive sites. However, charge‐trapping phenomena cannot justify the microsecond‐range EL decay observed in **TTH**‐OLETs, since the typical fingerprint of charge trapping in the EML, (i.e., a signal overshooting of the EL(t) at the end of the driving voltage pulse application [20, 21]) are absent in the EL(t) decay of **TTH**‐OLETs (Figure S4a,b). Figure 3b shows a comparison between the EL(t) of 5% and 15% doped **TTH**‐OLETs in a 25 µs‐wide time range. The major difference in EL(t) between the two devices is observed during the applied *V*_DS_ pulse: in 15% **TTH**‐OLETs the rise time of the EL signal is clearly slower than that of 5% **TTH**‐OLETs. Nevertheless, the nature of the excited state of **TTH** molecules, characterized by a CT character might induce a dynamic formation of localized internal field at guest sites that modulates (i.e., reduces) the rate of exciton formation. For this reason, a presence of higher density of **TTH** sites induces a slower EL(t) increase during *V*_DS_ bias application. Despite the fact that radiative mechanisms are dominated by a saturation process, the device steady‐state optical power is not limited by this EML doping level at 15% (Table 1).

Proceeding in the comparison between singlet diradicaloids and doublet radicals as emissive species in OLETs, we observed that (intensity‐averaged) lifetime constant is much shorter in **TTH**‐OLET than the corresponding radical‐OLET realized with **PyBTM‐(Me_2_Res)** at the same doping concentration (i.e., 0.7 ± 0.1 µs and 1.8 ± 0.1 µs, respectively): this evidence further corroborates that the exciton formation in **TTH**‐OLET is not driven by charge trapping at the guest molecules in the EML. Moreover, the relatively moderate photoresponse observed in organic phototransistors (OPTs) incorporating **TTH** molecules corroborates this hypothesis (Figure S2). We fabricated OPTs with **TTH** embedded in the bulk heterojunction comprising the photoactive layer to evaluate the charge trapping ability of the diradicaloid compound. Given the photosensitivity decrease of more than two orders of magnitude with respect to the case of radical molecules dispersed in the same matrix (approximately 4.5 × 10^2^ vs. 1 × 10^5^) [48], it is evident that **TTH** exhibits a negligible trapping capacity, significantly lower than that of the radical‐based molecules. Interestingly, the maximum EQE of 15% **TTH**‐based OLETs, that is, around 0.18% (Figure S2), is almost similar to that of the corresponding radical‐OLETs based on **PyBTM‐(Me_2_Res)**, that is, 0.20%: however, the EQE of **TTH**‐OLETs is almost invariant with respect to applied *V*_GS_ bias (apart from negligible fluctuations) in contrast with the radical‐OLET. The roll‐off of EQE is typically expected when charge‐exciton interaction is relevant even at low voltage bias such as in the case of exciton formation mediated by charge trapping. In the case of **PyBTM‐(Me_2_Res)**‐OLETs, we have demonstrated that the balance between the hole and electron densities was maximized in the active region of the device (with consequent increase of EQE) in virtue of the charge trapping at the emissive sites, corresponding to 7% of charge recombination efficiency 𝛾 in the EQE formula [49]. Given that bimolecular exciton formation by means of charge trapping at the **TTH** sites has been ruled out in EML of OLETs according to our experimental evidence, major and minority charge carriers are largely unbalanced in **TTH‐**OLET as expected for unipolar bilayer OLETs. Thus, we may infer that 𝛾 is inherently lower in TTH‐OLETs than in radical‐based counterpart, reaching a value of 7% as the upper limit achievable only in the best‐case scenario. As a consequence, the other factors in the EQE expression must be readjusted to guarantee the almost invariant EQE between **TTH**‐OLET and radical‐OLET experimentally determined. Recalling the EQE formula, (EQE = 𝜂_out_ × 𝜑_PLQY_ × 𝛾 × 𝜂_spin_) the factors, other than the charge balance 𝛾, are: (i) the outcoupling efficiency of photons (𝜂_out_), that is, typically considered independent from the OSC stack and assumed to be around 0.25 for device architectures similar to the one adopted here [16], (ii) the photoluminescence quantum yield (𝜑_PLQY_) of the EML that is measured independently, (iii) the spin statistical factor (𝜂_spin_) which reflects the multiplicity limit of radiatively recombining excitons. Specifically, considering the measured values of PLQY of the **TTH**‐based (0.35) and **PyBTM‐(Me_2_Res)** based (0.15) EML inserted into the EQE formula, it is evident that the 𝜂_spin_ must be larger than the 0.25 value expected for emission from purely singlet excitons. Following this argument, the effective spin statistical factor of **TTH** dyes in the EML can be inferred to be approximately ≥ 0.38, in order to reproduce the maximum EQE observed from the saturation transfer characteristics at source‐gate voltage of 79 V (Figure S2). The previous discussion emphasizes that several complex processes can occur in the active region of the OLET. Nevertheless, there is clear evidence that emissions occur from the **TTH** molecules. Furthermore, the analysis suggests that the spin statistical limit in devices based on **TTH** is larger than expected for a singlet‐spin emitting species.

Seeking a molecular‐level understanding of the mechanisms that can contribute to the generation of the **TTH** emissive state in the device, we carried out density functional theory (DFT) and time‐dependent density functional theory (TD‐DFT) calculations to map the lowest **TTH** excited states. The computed adiabatic energy differences, summarized in Figure S7, show that the lowest triplet state T_1_ lies very close to the ground electronic state, ca 0.2 eV, making highly unlikely the population of the SE state by TTA. In contrast, the calculations reveal an energy proximity between the SE state and the second triplet state, T_2_, dominated by the HOMO‐1 to LUMO excitation (Figure S8). Its minimum lies above the minimum of the bright SE state, with an adiabatic energy difference of ca 0.24 eV. A negative value for Δ*E*_S1‐T2_ promotes a fast RISC from the hot triplets to singlet [50]. In addition, computed spin–orbit coupling (SOC) integrals (Table S5) between the T_2_/SE states amount to ca. 11 cm^−1^, which are remarkable for an organic molecule and compatible with fast ISC rate constants [51]. The different orbital nature of the SE and T_2_ states justifies the sizable coupling. Thus, both energy proximity and SOCs suggest that RISC from T_2_ to SE can occur efficiently, as confirmed by TC‐SPC data of **TTH** when embedded in **TBPi** matrix (Figures S13 and S14). At the same time, the unusually large energy difference between T_1_ and T_2,_ and remarkably small T_1_→T_2_ TD‐DFT computed oscillator strength (0.008), disfavors radiative deactivation from T_2_. Two mechanisms can be invoked to account for the population of the hot triplet exciton of **TTH** in the device: the hole–electron recombination schematically represented in the left part of Figure 4, and the energy transfer from the lowest triplet state of **TPBi** in the blends, as shown in the right part of Figure 4. Notably, the T_2_ triplet of **TTH** lies below the lowest triplet state of **TPBi**, making energy transfer possible.

**FIGURE 4:**
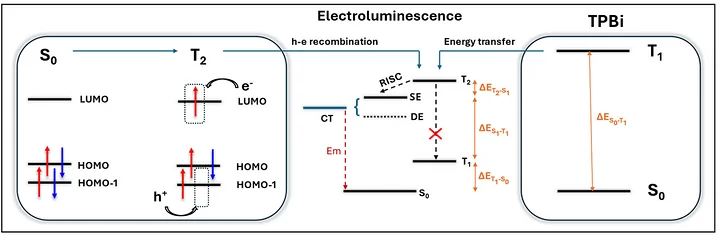
Schematic representation of the proposed hot‐exciton mechanism, supported by quantum‐chemical calculations and transient absorption experiments and involving the T_2_ and SE states of **TTH**. Plausible mechanisms leading to the population of T_2_ are also shown: (left) hole–electron recombination and (right) energy transfer from **TPBi** in the blends.

Furthermore, both the femtosecond and nanosecond transient absorption spectra of the **TPBi:TTH** film (Figure 5c,f) exhibit an additional spectral feature centered at ca. 640 nm that is absent in **TTH** solution (Figures S21 and S22) and in PMMA:TTH films (Figure 5a,d), where the transient response is dominated by the emissive singlet excited‐state absorption at ca. 580 nm. **TPBi** itself also exhibits a long‐lived transient absorption in this spectral region (Figure 5b,e), which is interpreted as due to population of its triplet state via intersystem crossing. The spectral position of the additional 640 nm feature in the **TPBi:TTH** film agrees with the calculated T_2_ excited‐state absorption of **TTH** (Figure S9), and its assignment is further supported by independent triplet‐sensitization experiments (Figures S16–S18) demonstrating efficient population of the **TTH** triplet manifold even when using sensitizers characterized by high triplet energy (e.g., phenanthrene, *E*_T_ = 2.93 eV). We therefore assign the additional 640 nm feature to excited‐state absorption involving higher triplet states of **TTH**. Together with the excitation‐dependent PL dynamics, these results support a host‐mediated population pathway involving the higher triplet manifold of **TTH** and subsequent RISC to the emissive state, facilitated by the substantial T_2_–S_1_ computed SOC (Table S5), in agreement with the proposed hot‐exciton mechanism.

**FIGURE 5:**
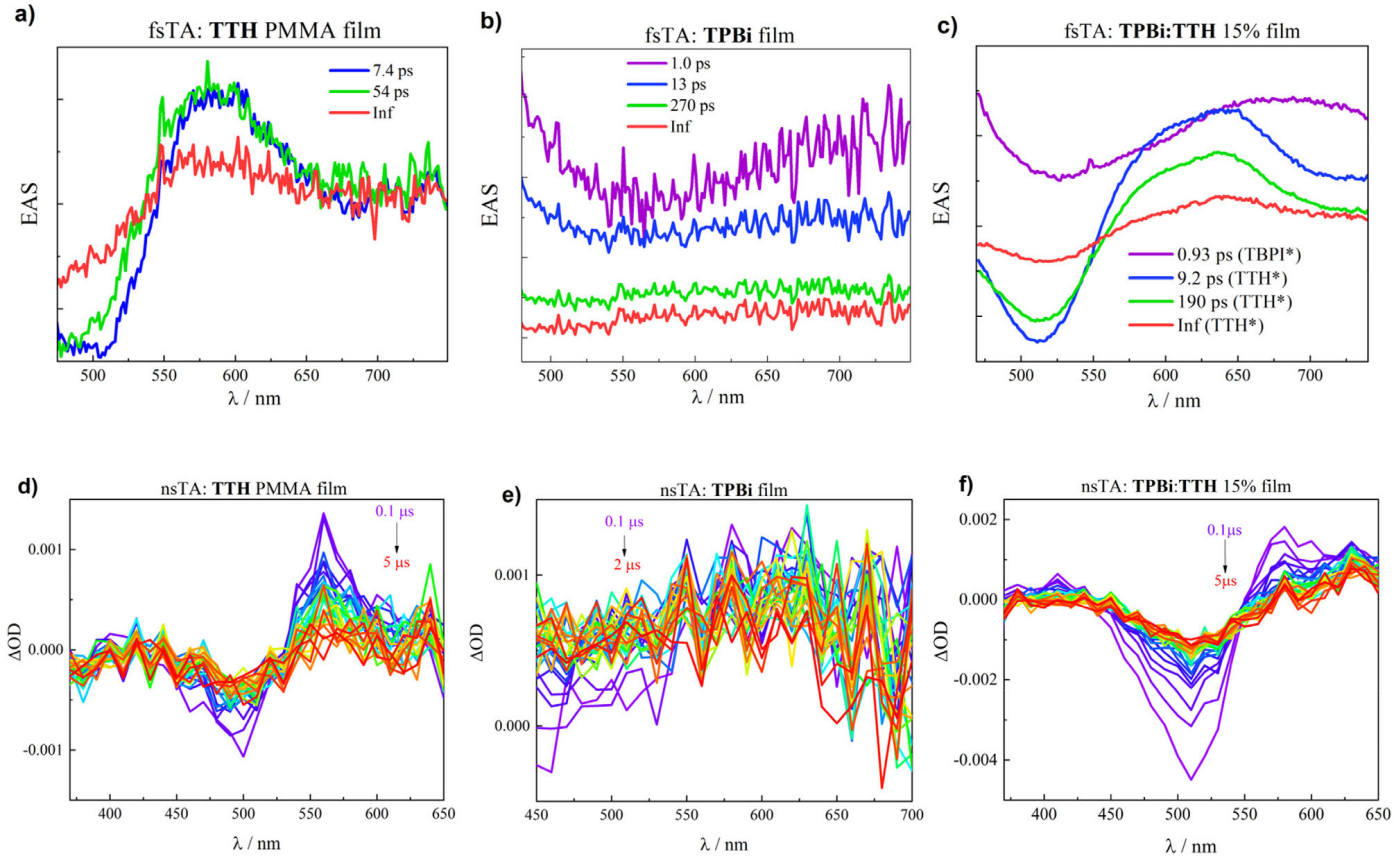
Evolution Associated Spectra (EAS) of the different transient species resulting from global fitting the femtosecond transient absorption (fsTA) results obtained upon 266 nm laser excitation for the **TTH** PMMA film, (a) **TPBi** film (b) and **TPBi**:**TTH** 15% film. (c) Nanosecond transient absorption (nsTA) spectra recorded upon 355 nm laser excitation of the **TTH** PMMA film, (d) **TPBi** film (e) and **TPBi**:**TTH** 15% film (f).

Based on the experimental and computational analysis of results, the proposed spin statistical factor of 0.38 should be regarded as a conservative lower‐bound estimate, with the actual value potentially being higher (since this value has been obtained considering 𝛾 = 7% on the whole range of the applied potential), justified by the involvement of a hot‐exciton mechanism. This mechanism resembles that observed in donor–acceptor systems with HLCT states, but is distinctive to the **TTH** diradicaloid, where a CT emitting state is coupled to a nearly degenerate triplet with different orbital nature.

## 3 Conclusions

Since the first demonstration that radical emitters can generate doublet excitons and bypass the spin‐statistical limit in OLEDs by Peng and coworkers [52], open‐shell molecules have emerged as a powerful platform for efficient electroluminescence. Here, we extend this concept beyond monoradicals and introduce singlet diradicaloids having a Thiele hydrocarbon backbone, as a new class of efficient emitters for electroluminescent transistors. We demonstrate, for the first time, that a small‐diradical‐character singlet diradicaloid operates as an efficient emitter in a field‐effect electroluminescent device, establishing the viability of diradicaloid photophysics within an OLET architecture. Despite the singlet ground state and the absence of thermally populated triplet states, the devices exhibit spin statistics exceeding the conventional 0.25 limit. Quantum‐chemical calculations, transient absorption experiments and time‐resolved electroluminescence measurements rule out TTA and instead support a hot‐exciton mechanism driven by the peculiar excited‐state alignment, in which a higher‐lying triplet T_2_ efficiently repopulates the emissive singlet *via* RISC. While hot‐exciton emitters are generally designed around donor–acceptor (push–pull) architectures, the use of singlet diradicaloids to promote hot‐exciton processes constitutes a fundamentally different strategy and, to the best of our knowledge, has not been reported previously.

At the same time, the absence of SOMOs mitigates charge trapping, reducing possible exciton formation via a bimolecular reaction mechanism. Extended comparative analysis of EL dynamics in working devices demonstrates that the different electronic landscape between diradicaloids and persistent radicals suggests that different strategies in device engineering can be implemented for increasing internal efficiency in electroluminescent devices. Specifically, the combination of enhanced spin conversion and relevant photoluminescence efficiency of **TTH** compound while controlling detrimental effects of charge trapping in working devices leads to remarkable EQE and significantly improved operational stability compared to conventional trityl radical emitters. Beyond the specific case of **TTH**, these results establish trityl‐based singlet diradicaloids as a distinct class of open‐shell emitters, where diradical character enables simultaneous control over spin dynamics and excited‐state relaxation pathways. This work highlights excited‐state engineering of open‐shell molecules as a promising strategy for overcoming intrinsic efficiency limitations in electroluminescent devices.

## Author Contributions

**Mario Prosa**: methodology, writing – original draft, writing – review and editing, formal analysis, investigation. **Eugenio Lunedei**: data curation, writing – original draft, writing – review and editing, formal analysis, investigation. **Michele Orza**: formal analysis, investigation. **Davide Mesto**: investigation, formal analysis. **Roberto Cantoni**: investigation, formal analysis, data curation. **Francesco Reginato**: investigation, formal analysis. **Angela Punzi**: funding acquisition, formal analysis, writing – review and editing, resources. **Arthur R. J. Barreto**: investigation, formal analysis. **Yasi Dai**: investigation, formal analysis. **Benedetta Carlotti**: investigation, resources, supervision, methodology, writing – review and editing, data curation. **Fabrizia Negri**: conceptualization, methodology, supervision, resources, writing – review and editing, funding acquisition, writing – original draft. **Stefano Toffanin**: funding acquisition, resources, supervision, methodology, conceptualization, writing – original draft, writing – review and editing. **Davide Blasi**: conceptualization, methodology, supervision, writing – original draft, writing – review and editing, funding acquisition.

## Conflicts of Interest

The authors declare no conflicts of interest.

## Supporting information




**Supporting File**: The authors have cited additional references within the Supporting Information.

## Data Availability

The data that support the findings of this study are available from the corresponding author upon reasonable request.
