# Supplementary material for: Beyond Radical Emitters: Hot‐Exciton Electroluminescence From Singlet Diradicaloids
Source: Angew Chem Int Ed Engl. 2026 Sep 22;65(45):e6379738. doi: 10.1002/anie.6379738 (PMC13725620; doi:10.1002/anie.6379738)
Supplement: Supplementary file 1 [file ANIE-65-e6379738-s001.docx]

**Supporting Information**

**Beyond Radical Emitters: Hot-Exciton Electroluminescence from Singlet Diradicaloids**

Mario Prosa^a⸶^, Eugenio Lunedei^a⸶^, Michele Orza^b^, Davide Mesto^c^, Roberto Cantoni,^[d]^ Francesco Reginato^a^, Angela Punzi^c^, Arthur R. J. Barreto^a^, Yasi Dai^b^, Benedetta Carlotti,^[d]^ Fabrizia Negri^b,e*^, Stefano Toffanin^a*^ and Davide Blasi^c*^

[a]	Dr. Mario Prosa, Dr. Francesco Reginato, Dr. Eugenio Lunedei, Dr. Arthur R.J. Barreto, Dr. Stefano Toffanin
Istituto per lo Studio dei Materiali Nanostrutturati, Consiglio Nazionale delle Ricerche
Via P. Gobetti 101, 40129, Bologna, Italy

E-mail: stefano.toffanin@cnr.it

[b]	Michele Orza, Dr. Yasi Dai, Prof. Fabrizia Negri
Dipartimento di Chimica “Giacomo Ciamcian”, Università di Bologna,
Via P. Gobetti 85, 40129, Bologna, Italy
E-mail: [fabrizia.negri@unibo.it](mailto:fabrizia.negri@unibo.it)

[c]	Dr. Davide Mesto, Prof. Angela Punzi, Dr. Davide Blasi
Dipartimenti di Chimica, Università degli Studi di Bari Aldo Moro
Via E. Orabona 4, 70125, Bari, Italy
E-mail: [davide.blasi@uniba.it](mailto:davide.blasi@uniba.it)

[d]	Roberto Cantoni, Prof. Benedetta Carlotti
Department of Chemistry, Biology and Biotechnology
University of Perugia
Via Elce di Sotto 8, 06123 Perugia, Italy

[e]	Prof. Fabrizia Negri
Center for Chemical Catalysis—C3
Alma Mater Studiorum—Università di Bologna
Via P. Gobetti 85, 40129 Bologna, Italy
and INSTM UdR Bologna, Italy

⸶ These authors contributed equally to this work

**Contents**

General Methods…………………………………………………………………………….S2

Steady-state optoelectronic characterization of TTH-based OLETs…………………………………...……...………………………………….S5

Transient electroluminescence of TTH-based OLETs………………………...S8

Optoelectronic characterization of TTH-based OPT…………...…………………………………………………………………….S9

Computational details…..………………………………………………………………..………..S10

Time Correlated Single Photon Counting..…………………………………………………………………….…..S16

Sensitization Experiments….…………………………………………………………………..S19

Nanosecond and Femtosecont transient absorption….……………………………………………………………………..S23

References……………………………………………………………………….S26

**General Methods**

**TTH synthesis**

Thiele hydrocarbon **TTH** was synthesized according to a previously reported procedure.^[1]^

**Devices Fabrication**

All the devices were fabricated in bottom-gate/top-contact configuration on 25mm × 25mm transparent glass/ITO substrates. In the case of OLETs devices, 450 nm thick PMMA layer was deposited by spin-coating as gate dielectric in according to the literature procedure.^[2]^ TTH-OLETs structure were composed of i) 40 nm of n-type semiconductor P13 (Sigma–Aldrich) thermally sublimed at 0.1 Å sec−1 ii) 60 nm thick host–guest EML of TPBi (Ossila) (host) and TTH (guest) at different concentrations, i.e. 5%, 15% and 22.5% sublimed maintaining the guest deposition rate constant at 0.1 Å sec^−1^ while decreasing the deposition rate of the host from 2 to 0.6 to 0.6 Å sec^−1^, respectively. In the case of 15% concentration, the EML thickness was varied to 30 and 45 nm. The HIL consisted of 15 nm of CBP (American Dye Source, Inc.) sublimed at 0.1 Å sec^−1^. The organic stack was covered by 80 nm thick gold source and drain electrodes deposited by thermal sublimation at 0.5 Å sec^−1^. The same deposition conditions of gold source were maintained also in the case of asymmetric electrode configuration. The resulting devices presented the following geometrical characteristics: 70 μm channel length (L), 12 mm channel width (W) and 500 μm wide source and drain electrodes.

In the case of OPT fabrication, the devices adopted a bottom-gate, middle-contact configuration (please consider the sketch of the device reported in Figure S5a. The glass/ITO substrates were cleaned in an ultrasonic bath in acetone twice for 10 min, 2-propanol for 10 min, and then dried with argon flow. All substrates underwent surface treatment with oxygen plasma prior to polymer film deposition. The ferroelctric dielectric layer was obtained by filtering a solution of P(VDF-TrFE-CFE) (70:30:8.5) dispersed in cyclopentanone through a 0.45 m PTFE filter and then spin-coating the solution at 4000 rpm for 90 s and annealed in a nitrogen atmosphere in a glovebox on a hot plate at 110 °C for about 30 min to remove the solvent (Ci = 76 nF/cm^2^ and thickness = 500 nm). Then, the hole-transporting semiconducting layer was obtained by spin-coating the DPP-DTT:C6Si chlrobenzene solution in controlled atmosphere on the dielectric at 1000 rpm for 60 s. DPP-DTT was purchased by Arkema and C61 by Sigma Aldrich. After exposure to air for about 1 h, the polymerfilms were returned in controlled atmosphere and annealed at 90 °C overnight and then at 200 °C for 10 min. The top contact source/drain electrodes were deposited by the thermal sublimation of Ag (70 nm thickness) using a metal shadow mask (100 μm channel length and 5000 μm channel width). Afterward, the photoactive bulk heterojunction layer was obtained by filtering a chlroform solution of DPP-DTT:TTH = 1:3 in weight ratio through a 0.45 m PTFE filter, and then by spin-coating the solution at 1000 rpm for 60 s, and then placed on a hot plate for 10 min at 135 °C.

**Optoelectronic Characterization of optoelectronic devices**

The electrical and optoelectronic measurements of OLETs were performed in an inert atmosphere inside a nitrogen-filled glovebox using a standard SUSS probe station coupled to a B1500A Agilent semiconductor device analyzer. The light output was measured at the bottom side of the substrates with a silicon photodiode (a sensitivity of 0.38 A W^−1^ at 600 nm) directly in contact with the devices. OLET steady state EL spectrum was obtained collecting the signal with a Hamamatsu PMA optical multichannel analyzer. The field-effect mobility in the saturation regime (μ) was calculated using the equation I_DS_ = (W/2L)C_i_μ(V_GS_ – V_Th_)^2^, where C_i_ is the capacitance per unit area of the insulating layer and V_Th_ is the threshold voltage extracted from the square root of the drain current (I_DS_^1/2^) versus gate voltage (V_GS_) characteristics.

The electrical and optoelectronic measurements of OPTs were performed under dark conditions inside a glovebox using a standard SUSS probe station coupled with a B1500A Agilent semiconductor device analyzer. The same equipment was used to measure the OPT characteristics under NIR illumination with an LED source (Thorlabs), which peaked at 770 nm with an intensity modulable from 2 mW/cm^2^ to lower values by using neutral optical density filters. The LED is driven using a Keithley 236 source measurement unit with a voltage input of 50 mA. The optical power density of the output illumination was measured using a calibrated Si photodiode.

**Optical characterization of TTH-based films**

PL spectra were obtained with a Hamamatsu PMA optical multichannel analyzer by optically exciting the films comprising TPBI:TTH blends deposited on quartz at 325 nm to verify possible energy transfer mechanism from TPBI matrix to TTH guest molecules. The PLQY measurements were obtained with a FS5-SS Hamamatsu steady state spectrometer with the correlated integrating sphere module.

**Time-resolved EL characterisation**

In time-resolved EL measurements OLET gate electrode was biased by a Keithley 2400 SMU at a fixed voltage, whereas an Agilent 8114A generator was used for applying pulsed V_DS_ bias. The output low of both sources was common grounded. Two V_DS_ pulse widths were applied, i.e. 1 and 5 μs, whereas the pulse repetition rates were 108 and 10 kHz, respectively. Photons emitted from the device were collected using an f/4, 300 mm, Acton Research Spectra Pro SP-2300i triple-turret monochromator with a Hamamatsu H7422-20 Peltier-cooled photosensor module as the detector. EL transients were measured by means of PicoQuant TimeHarp-100 and PicoQuant NanoHarp-250 electronic boards synchronously driven by the pulse generator.

**Experimental Details for the Time-Correlated Single Photon Counting and Transient Absorption Measurements**

Fluorescence kinetics were acquired using the time-correlated single photon counting (TC-SPC) method through the Edinburgh Instrument 199S spectrofluorometer, equipped with a LED sources centered at 263 nm, 370 nm and 460 nm with a 0.3 ns temporal resolution.

Nanosecond transient absorption studies were conducted by laser flash photolysis (Edinburgh LP980) upon excitation at 355 nm (third harmonic of a Quanta-Ray/ INDI Nd:YAG laser, Spectra Physics) with nanosecond time resolution (pulse width 7 ns and laser energy < 1 mJ per pulse) coupled with a PMT for signal detection. The excitation at 355 nm was the pump-pulse while a pulsed xenon lamp was used to probe the absorption properties of the produced excited states. Sensitization experiments were carried out by energy transfer from Phenanthrene, Anthracene, Tetracene, Rubrene, Pentacene to the studied compound in dichloromethane (DCM), chloroform (CHCl_3_) or toluene (Tol). All measurements in solution were performed by purging the sample with pure molecular nitrogen. The eventual degradation of TTH was controlled by measuring the absorption spectra before and after the experiment, and no degradation was found for this compound.

The experimental setup for ultrafast transient absorption experiments has been previously reported.^[3,4]^ The 400 nm and 266 nm excitation pulses (ca. 60 fs) were generated as the second and third harmonic of an amplified Ti:Sapphire laser system (Spectra Physics). The Helios transient absorption spectrometer (Ultrafast Systems) is characterized by a temporal resolution of about 150 fs and a spectral resolution of 1.5 nm. A small portion of the 800 nm light passes through an optical delay line (time window of 3200 ps) and is focused onto a Sapphire crystal (2 mm thick) to generate a white-light in the 450-800 nm spectral range (probe pulse). All measurements were carried out under the magic angle relative polarization condition. The solutions were stirred and the films were kept under constant movement during the experiments to avoid photoproduct interferences. The experimental 3D data matrices were firstly analyzed performing the Global Analysis by Surface Xplorer PRO (Ultrafast Systems) software, and successively through GloTarAn software.^[5]^

**Steady-state optoelectronic characterization of TTH-based OLETs**

**a)**

**b)**

**c)**

**Figure S1.** TTH-OLETs n-type curves: a) saturation transfer curve for the OLET doped with TTH at 5 %; b) multiple output curve for the OLET doped with TTH at 15 %; c) multiple output curve for the OLET doped with TTH at 5 %.

**Table S1.** Summary of the figures of merit of the TTH-OLET with EML doped at 22,5 %.

| **EML**  **PLQY** | **μ ^a)^**  **[cm^2^ V^-1^ s^-1^]** | **V_th_ ^a)^**  **[V]** | **I_DS_ ^b)^**  **[μA]** | **EL ^b)^**  **[nW]** | **EQE ^b)^**  **[%]** |
| --- | --- | --- | --- | --- | --- |
| 0,16 | 2,5 ∙ 10^-2^ | 16 | 120 | 95 | 0,05 |

^a)^ calculated from saturation transfer curves in the saturation regime; ^b)^ at V_GS_ = V_DS_ = 100 V

EQE (%)

V_GS_ (V)

**Figure S2.** EQE of OLETs, including a 60 nm-thick EML doped with 15% of TTH, calculated from saturation transfer characteristics under positive polarization at V_DS_ = 100 V.

**a)**

**b)**

**c)**

**45 nm**

**30 nm**

**Figure S3.** Saturation transfer curves (V_DS_ = 100V) of OLETs doped with TTH at 15 %: a) EML thickness: 45 nm; b) EML thickness: 30 nm; c) EML thickness: 60 nm; asymmetric source and drain electrodes.

**Table S2.** Figures or merit of OLETs doped with TTH at 15 % as a function of the EML thickness and electrodes layout.

| **ELM thickness [nm]** | **μ ^a)^**  **[cm^2^ V^-1^ s^-1^]** | **V_th_ ^a)^**  **[V]** | **I_DS_ ^b)^**  **[μA]** | **EL ^b)^**  **[nW]** | **EQE ^b)^**  **[%]** | **Brightness ^b)^**  **[cd m^-2^]** |
| --- | --- | --- | --- | --- | --- | --- |
| 30 | 7,9 E-2 | 5,0 | 415 | 56 | 0,008 | - |
| 45 | 4,8 E-2 | 2,3 | 250 | 87 | 0,02 | - |
| 60 | 1,7 E-2 | 6,4 | 85 | 217 | 0,15 | 0,87 |
| 60 - asymmetric electrodes | 2,0 E-1 | 4,6 | 1060 | 857 | 0,05 | 7,63 |
|  |  |  |  |  |  |  |

^a)^ calculated from saturation transfer curves in the saturation regime; ^b)^ at V_GS_ = V_DS_ = 100 V

**Transient electroluminescence of TTH-based OLETs**

**a)**

**b)**

**Figure S4.** 100 μs wide time range normalized semi-log plot of EL(t) of 5% (panel a, blue line) and 15% (panel b, green line) doped TTH-OLETs by applying rectangular V_DS_ pulses (width = 5 μs, 10 kHz rep. rate). Panel a.inset and panel b.inset: Normalized EL(λ, Δt) spectra collected within various time intervals Δt (W0, W1, W2, and W3) as indicated on the x-axis of the main panels. Regardless the TTH doping concentration, all spectra showed identical characteristics that are associated with the emission from TTH molecules.

**Table S3.** Decay times (τ_1_, τ_2_ and τ_3_) and fractional intensities (W_1_, W_2_ and W_3_) of transient EL of 5% and 15% doped TTH-OLETs at different V_DS_ = 80 V pulse widths and at constant V_GS_ = 80 V.

| **V_DS_ pulse**  **width** | **TTH**  **Conc %** | **τ_1_**  **(μs)** | **W_1_**  **(%)** | **τ_2_**  **(μs)** | **W_2_**  **(%)** | **τ_3_**  **(μs)** | **W_3_**  **(%)** | **τ_AVG_**  **(μs)** |
| --- | --- | --- | --- | --- | --- | --- | --- | --- |
| 1 μs | 5 % | 1,07 ± 0,04 | 55,99 | 0,173 ± 0,008 | 31,50 | 0,032 ± 0,003 | 12,50 | 0,7 ± 0,1 |
|  | 15 % | 1,15 ± 0,04 | 56,65 | 0,149 ± 0,006 | 34,25 | 0,016 ± 0,002 | 9,11 | 0,7 ± 0,1 |
| 5 μs | 5 % | 8 ± 1 | 54,68 | 1,0 ± 0,2 | 34,85 | 0,14 ± 0,07 | 10,47 | 4,7 ± 0,1 |
|  | 15 % | 0,149 ± 0,008 | 37,26 | 12,7 ± 0,8 | 31,90 | 1,6 ± 0,1 | 30,84 | 4,6 ± 0,1 |

**Optoelectronic characterization of TTH-based OPT**

**a)**

**b)**

**c)**

**d)**

**V_GS_ (V)**

**I_DS_ (A)**

**Figure S5**. (a) Cross-sectional view of OPTs with a bottom-gate middle-contact configuration. (b) Absorption spectra of DPP-DTT:TTH = 1:3 BHJ and pristine DPP-DTT thin films. (c) Transfer curves at V_DS_ = −8 V in the dark and under 2 mW/cm^2^ of NIR light, peak at 780nm, and (d) its corresponding sensitivity curve.

**Computational details**

Quantum-chemical calculations were carried out on **TTH** and **TPBi** to investigate their excited state proximity. To this end, ground and excited state geometries were optimized. The optimized ground state geometries of **TTH** and **TPBi** were determined at M06-2X/def2-SVP level of theory including the D3 version of Grimme’s dispersion.^[6]^ Vertical excitations energies were evaluated at TD-M06-2X level with the same basis set and D3 dispersion. Excited state geometries were obtained at TD-M06-2X-D3/def2-SVP level or with UM06-2X-D3/def2-SVP. Our previous investigations have shown that the M06-2X functional provides an accurate description of the bright singlet excited state SE of TTH. Specifically, the calculated absorption and emission energies of TTH agree with experiment to within 0.2/0.3 eV.

The optimized structures of the lowest triplet and second lowest triplet of **TTH** were determined and used, along with those of the ground and SE states (see Figure S6), to calculate adiabatic energy differences collected in Figure S7. The DFT and TD-DFT calculations were complemented by DFT-MRCI computations, a method that accounts for both dynamic and static electron correlation,^[7]^ to obtain adiabatic excitation energies. The resulting DFT-MRCI energy differences are reported in Table S4 alongside the corresponding TDDFT values for comparison.

The transient T_2_🡪 T_n_ absorption spectra (Figure S9) were calculated using TD-DFT within the Tamm–Dancoff approximation (TDA).^[8]^ To simulate the evolution of the transient absorption signal, the spectra were computed for a series of geometries interpolating between the ground-state equilibrium structure and the equilibrium geometry of the T_2_ state.Spin-orbit coupling (SOC) integrals were calculated with the spin–orbit mean-field (SOMF) method, with one-center approximation applied to the exchange term, (SOMF(1X)).^[9,10]^ Relativistic corrections were included with the zeroth order regular approximation (ZORA)^[11]^ using the ZORA-def2-TZVP basis set. Excited state energies were determined at TDA-M06-2X level. The SOC and the TDA-DFT transient absorption spectra were computed using ORCA 6.0.1,^[12]^ while all DFT and TD-DFT calculations were performed using the Gaussian 16 suite of programs.^[13]^

**Figure S6. TTH**: Optimized geometries of **TTH** in different electronic states. From M06-2X-D3/def2-SVP and TD-M06-2X-D3/def2-SVP calculations.

**Figure S7.** Schematic representation of the adiabatic energy differences between pairs of electronic states of **TTH**, showing the proximity of T_2_ to SE, and of **TPBi**, showing that the T_2_ state of **TTH** is energetically accessible by energy transfer from the T_1_ state of **TPBi**. From TD-M06-2X-D3/def2SVP and M06-2X-D3/def2SVP calculations.

**Figure S8.** Orbital nature of the lowest singly-excited states of **TTH**. From TD-M06-2X-D3/def2SVP calculations.

**Figure S9. TTH**: T_2_🡪T_n_ transient absorption spectra computed for TTH. From TDA-M06-2X/def2-SVP calculations. (Left, bottom) Spectrum computed @ the equilibrium geometry of the ground electronic state. (Left, top) Spectrum computed @ the equilibrium geometry of the T_2_ state. (Right) The spectra are computed for a series of geometries interpolating between the ground-state equilibrium structure (bottom) and the equilibrium geometry of the T_2_ state (top).

**Figure S10.** Optimized geometries of **TPBi** in the ground and lowest triplet electronic states. From M06-2X-D3/def2-SVP and TD-M06-2X-D3/def2-SVP calculations.

**Table S4.** Adiabatic transition energies (eV) computed for TTH at TD-M06-2X/def2SVP and DFT-MRCI level.

|  |  | **ΔE(SE-S_0_)** | **ΔE(T_2_-S_0_)** |
| --- | --- | --- | --- |
| **TD-DFT** |  | 2.18 | 2.42 |
| **DFT-MRCI** |  | 1.92 | 2.47 |

**Table S5.** Spin orbit coupling(SOC) values, in cm^-1^, computed at the ground state geometry of **TTH** in Cartesian basis. The real and imaginary parts are in parenthesis.

|  | **ZORA-def2-TZVP/SARC-ZORA-TZVP @ geo PCM-B3LYP** | | | |
| --- | --- | --- | --- | --- |
|  |  | **X(Re, Im)** | **Y (Re, Im)** | **Z (Re, Im)** |
| **TTH** | SE/T_2_ | (0.00 , 0.00) | (0.00 , -11.13) | (0.00 , 0.00) |

**Time Correlated Single Photon Counting**

**Figure S11**. Fluorescence decay kinetics of TTH in chloroform obtained via TC-SPC upon 460 nm excitation.

**Figure S12**. Fluorescence decay kinetics of TTH in PMMA film obtained via TC-SPC upon 460 nm excitation.

**Figure S13**. Fluorescence decay kinetics of TPBI-TTH 15% film obtained via TC-SPC upon 263 nm excitation.

**Figure S14**. Fluorescence decay kinetics of TPBI-TTH 15% film obtained via TC-SPC upon 370 nm excitation.

**Figure S15**. Fluorescence decay kinetics of TPBI-TTH 15% film obtained via TC-SPC upon 460 nm excitation.

**Sensitization Experiments**

**Figure S16**. Sensitization experiment using phenanthrene as triplet energy donor and TTH as energy acceptor: triplet spectrum of phenanthrene in CHCl_3_ (figure above on the left); triplet spectrum of phenanthrene in the presence of TTH in CHCl_3_ (figure above on the right); decay kinetic at 430nm for phenanthrene (figure below on the left); decay kinetic at 430nm for phenanthrene in the presence of TTH (figure below on the right). Evidence for the TTH T_1_ absorption at ca. 350 nm is observed in the transient spectra recorded for the mixed solution at long delays after excitation, consistent with the theoretical predictions.

**Figure S17**. Sensitization experiment using anthracene as triplet energy donor and TTH as energy acceptor: triplet spectrum of anthracene in DCM (figure above on the left); triplet spectrum of anthracene in the presence of TTH in DCM (figure above on the right); decay kinetic at 430nm for anthracene (figure below on the left); decay kinetic at 430nm for anthracene in the presence of TTH (figure below on the right).

**Figure S18**. Sensitization experiment using tetracene as triplet energy donor and TTH as energy acceptor: triplet spectrum of tetracene in CHCl_3_ (figure above on the left); triplet spectrum of tetracene in the presence of TTH in CHCl_3_ (figure above on the right); decay kinetic at 465nm for tetracene (figure below on the left); decay kinetic at 465nm for tetracene in the presence of TTH (figure below on the right).

**Figure S19**. Sensitization experiment using rubrene as triplet energy donor and TTH as energy acceptor: decay kinetic at 480nm for rubrene in CHCl_3_ (figure on the left); decay kinetic at 480nm for rubrene in the presence of TTH in CHCl_3_ (figure on the right).

**Figure S20**. Sensitization experiment using pentacene as triplet energy donor and TTH as energy acceptor: decay kinetic at 505nm for pentacene in toluene (figure on the left); decay kinetic at 505nm for pentacene in the presence of TTH in toluene (figure on the right).

**Table S6**. Quenching rate constants for the sensitization experiments: E_T_ = triplet energy of the sensitizer; τ_0_ =triplet lifetime of the sensitizer; τ = quenched sensitizer triplet lifetime in presence of acceptor (TTH); c_TTH_ = concentration of acceptor TTH; k_q_ = quenching rate constant, calculated by $\frac{\tau_{0}}{\tau}=1+k_{q}\tau_{0}c_{TTH}$ Diffusional rate constant for dichloromethane k_diff_ = 1.5×10^10^ M^−1^ s^−1^ at 298 K; for chloroform k_diff_ = 1.2×10^10^ M^−1^ s^−1^ at 298 K; for toluene k_diff_ = 1.1×10^10^ M^−1^ s^−1^ at 298 K.

| **Sensitizer** | **Solvent** | **E_T_ / eV** | **τ_0_ / μs** | **τ / μs** | **c_TTH_ / M** | **k_q_ / M^-1^s^-1^** |
| --- | --- | --- | --- | --- | --- | --- |
| Phenanthrene | CHCl_3_ | 2.92 | 91.8 | 17.9 | 1.2·10^-5^ | 3.6·10^9^ |
| Anthracene | DCM | 1.85 | 22.4 | 7.85 | 1.6·10^-5^ | 5.2·10^9^ |
| Tetracene | CHCl_3_ | 1.27 | 53.6 | 6.80 | 2.8·10^-5^ | 4.6·10^9^ |
| Rubrene | CHCl_3_ | 1.14 | 57.6 | 35.0 | 8.4·10^-6^ | 1.3·10^9^ |
| Pentacene | Tol | 0.77 | 29.1 | 23.7 | 1.1·10^-5^ | 7.1·10^8^ |

**Nanosecond Transient Absorption**

**Figure S21**. Nanosecond transient absorption spectra of TTH in chloroform upon 355 nm laser excitation.

**Femtosecond Transient Absorption**

**Figure S22**. Femtosecond transient absorption of TTH in chloroform upon 400 nm laser excitation. A) tridimensional data matrix ΔA as a function of wavelength and time; B) time resolved absorption spectra at different delays after photoexcitation; C) kinetics at representative wavelengths; D) Evolution Associated Spectra (EAS) and lifetimes of the transient species revealed through Global Analysis.

**Figure S23**. Femtosecond transient absorption of PMMA film of TTH upon 266 nm laser excitation. A) tridimensional data matrix ΔA as a function of wavelength and time; B) time resolved absorption spectra at different delays after photoexcitation; C) kinetics at representative wavelengths; D) Evolution Associated Spectra (EAS) and lifetimes of the transient species revealed through Global Analysis.

**Figure S24**. Femtosecond transient absorption of the TPBI film upon 266 nm laser excitation. A) tridimensional data matrix ΔA as a function of wavelength and time; B) time resolved absorption spectra at different delays after photoexcitation; C) kinetics at representative wavelengths; D) Evolution Associated Spectra (EAS) and lifetimes of the transient species revealed through Global Analysis.

**Figure S25**. Femtosecond transient absorption of the TPBI-TTH 15% film upon 266 nm laser excitation. A) tridimensional data matrix ΔA as a function of wavelength and time; B) time resolved absorption spectra at different delays after photoexcitation; C) kinetics at representative wavelengths; D) Evolution Associated Spectra (EAS) and lifetimes of the transient species revealed through Global Analysis.

**Table S7**. Lifetimes and assignements of the components revealed upon Global Analysis of the femtosecond transient absorption data.

| Sample | Excitation | Medium | τ / ps | Assignment |
| --- | --- | --- | --- | --- |
| **TTH** | 400 nm | CHCl_3_ | 0.60 | Solv. |
|  |  |  | 4.1 | SE |
|  |  |  | 96 | DE hot |
|  |  |  | 26000 | DE |
| **TTH** | 266 nm | PMMA film | 7.4 | SE |
|  |  |  | 54 | DE hot |
|  |  |  | Inf | DE |
| **TPBi** | 266 nm | film | 1.0 | VC |
|  |  |  | 13 | VC |
|  |  |  | 270 | S_1_ |
|  |  |  | Inf | T_1_ |
| **TPBi**+**TTH** | 266 nm | film | 0.93 | TPBI* |
|  |  |  | 9.2 | TTH* |
|  |  |  | 190 | TTH* |
|  |  |  | Inf | TTH* |

**References**

[1] A. Punzi, Y. Dai, C. N. Dibenedetto, E. Mesto, E. Schingaro, T. Ullrich, M. Striccoli, D. M. Guldi, F. Negri, G. M. Farinola, D. Blasi, *J. Am. Chem. Soc.* **2023**, *145*, 20229–20241.
[2] M. Zambianchi, E. Benvenuti, C. Bettini, C. Zanardi, R. Seeber, D. Gentili, M. Cavallini, M. Muccini, V. Biondo, C. Soldano, G. Generali, S. Toffanin, M. Melucci, J Mater Chem C. Mater 2016, 4, 9411.
[3] Y. Rout, C. Montanari, E. Pasciucco, R. Misra, B. Carlotti, *J. Am. Chem. Soc.* **2021**, *143*, 9933–9943.
[4] M. Alebardi, C. Munzone, E. Sorbelli, A. Grasso, L. Mencaroni, F. Elisei, C. G. Fortuna, A. Spalletti, C. Bonaccorso, B. Carlotti, *Adv. Fun. Mat.,* **2024**, *34*, 2403706
[5] J. J. Snellenburg, S. P. Laptenok, R. Seger, K. M. Mullen, I. H. M. van Stokkum, *J. Stat. Soft.* **2012**, *49*, DOI 10.18637/jss.v049.i03.
[6] S. Grimme, J. Antony, S. Ehrlich and H. Krieg, *J. Chem. Phys.*, **2010**, 132, 154104
[7] Marian CM, Heil A, Kleinschmidt M. *WIREs Comput Mol Sci*. **2019**; 9:e1394
[8] S. Hirata, M. Head-Gordon, *Chem. Phys. Lett*., **1999**, 314, 291-299.
[9] B. de Souza, G. Farias, F. Neese and R. Izsák, *J. Chem. Theory Comput.,* **2019**, 15, 1896–1904.
[10] F. Neese, *J. Chem. Phys.,* **2005**, 122, 034107.
[11] E. van Lenthe, J. G. Snijders and E. J. Baerends, *J. Chem. Phys.,* **1996**, 105, 6505–6516.
[12] F. Neese, F. Wennmohs, U. Becker and C. Riplinger*, J. Chem. Phys.,* **2020**, 152, 224108; F. Neese, Wiley *Interdiscip. Rev.: Comput. Mol. Sci*., **2025**, 15, 2, e70019.
[13] M. J. Frisch, G. W. Trucks, H. B. Schlegel, G. E. Scuseria, M. A. Robb, J. R. Cheeseman, G. Scalmani, V. Barone, G. A. Petersson, H. Nakatsuji, X. Li, M. Caricato, A. V. Marenich, J. Bloino, B. G. Janesko, R. Gomperts, B. Mennucci, H. P. Hratchian, J. V. Ortiz, A. F. Izmaylov, J. L. Sonnenberg, D. Williams-Young, F. Ding, F. Lipparini, F. Egidi, J. Goings, B. Peng, A. Petrone, T. Henderson, D. Ranasinghe, V. G. Zakrzewski, J. Gao, N. Rega, G. Zheng, W. Liang, M. Hada, M. Ehara, K. Toyota, R. Fukuda, J. Hasegawa, M. Ishida, T. Nakajima, Y. Honda, O. Kitao, H. Nakai, T. Vreven, K. Throssell, J. A. Montgomery, Jr., J. E. Peralta, F. Ogliaro, M. J. Bearpark, J. J. Heyd, E. N. Brothers, K. N. Kudin, V. N. Staroverov, T. A. Keith, R. Kobayashi, J. Normand, K. Raghavachari, A. P. Rendell, J. C. Burant, S. S. Iyengar, J. Tomasi, M. Cossi, J. M. Millam, M. Klene, C. Adamo, R. Cammi, J. W. Ochterski, R. L. Martin, K. Morokuma, O. Farkas, J. B. Foresman and D. J. Fox, Gaussian 16, Rev. C. 01.
